# Arrhythmia Diagnosis Following an ICD Shock: Reply

**Published:** 2002-01-01

**Authors:** Roy M John

Dr. Levine's comments [1] are greatly appreciated. Most modern ICDs have arrhythmia discrimination algorithms that have variable success in withholding inappropriate therapy. It must be borne in mind that enhancing specificity for ventricular arrhythmia detection can entail a loss of sensitivity. Accordingly, all algorithms necessarily err on the side of safety and will withhold therapy for only brief period of time. A persistent high rate will eventually be treated as a ventricular arrhythmia and can result in unnecessary and repetetive shocks. Hence the importance of aggressive management of supraventricular arrhythmias in these patients cannot be overemphasized.

The patient presented in the case study [2]  had an older generation single chamber ICD. Although, the provocation of ventricular tachycardia by anti-tachycardia pacing led to an ICD shock and termination of the atrial arrhythmia in this particular instance, atrial arrhythmias are often not terminated by ICD shocks delivered between an RV lead and the generator can. Further therapies with repeated induction of VT could have resulted. In patients with severe LV dysfunction or ischemia, such arrhythmias have a potential for progression to intractable ventricular fibrillation and death.

The modern dual chamber detection algorithms have significantly improved specificity for ventricular arrhythmia detection. I am in total agreement with Dr. Levine regarding the programming of monitoring zones in the early post implant period. When done correctly, such programming can provide a great deal of information on arrhythmia characteristics and accuracy of discrimination algorithms.
